# A Woman with Black Beads in Her Stomach: Severe Gastric Ulceration Caused by Yttrium-90 Radioembolization

**DOI:** 10.1155/2018/1413724

**Published:** 2018-04-15

**Authors:** Indu S. Voruganti, James L. Godwin, Alyn Adrain, Edward Feller

**Affiliations:** ^1^The Warren Alpert Medical School of Brown University, Providence, RI, USA; ^2^Jefferson University Hospitals, Philadelphia, PA, USA; ^3^Division of Medical Education, The Warren Alpert Medical School of Brown University, Providence, RI, USA

## Abstract

Radioembolization (RE) is a selective internal radiation therapy (SIRT) delivering targeted, high-dose, intra-arterial radiation directly to the vascular supply of liver tumors. Complications can occur due to aberrant deposition or migration of radiation microspheres into nontarget locations, including normal hepatic parenchyma, lungs, pancreas, and upper gastrointestinal (UGI) tract. We report a case of gastric ulcers due to yttrium-90 (^90^Y) seed migration to the stomach to alert clinicians to this rare cause of gastric injury. A 57-year-old woman with stage IV breast cancer with liver and lung metastases presented to the hospital with 2 months of worsening nausea and vomiting. Two months prior, she had received SIRT with ^90^Y microspheres without complications. Upper GI endoscopy showed diffuse gastritis and extensive antral ulceration. Biopsies revealed black, spherical foreign bodies, consistent with ^90^Y microspheres, documenting radiation injury. Radiation-induced UGI ulceration is caused by direct radiation injury from beta-radiation. Delay in diagnosis may be due to the nonspecificity of symptoms and temporal delay of symptom onset from SIRT, which was 2 months in our patient. Also, complaints may be attributed erroneously to adjuvant chemotherapy or widespread metastatic disease. Clinicians must consider radiation-associated toxicity in any SIRT-treated patient developing abdominal symptoms.

## 1. Introduction

Radioembolization (RE) is a selective internal radiation therapy (SIRT) technique which delivers targeted, precise, high-dose, intra-arterial radiation directly to the vascular supply of liver tumors [1]. A common SIRT protocol uses beta-emitting yttrium-90 (^90^Y) microspheres, infused by intra-arterial catheters via the hepatic arteries. The procedure's rationale is that primary and metastatic liver tumors are vascularized by arterial blood flow, whereas normal hepatocytes obtain their blood supply from the portal venous network. The procedure aims to spare normal, contiguous hepatic parenchyma, and adjacent viscera [2, 3].

However, complications can occur, predominantly due to aberrant deposition or migration of radiation microspheres into unintended nontarget locations, including normal hepatic parenchyma, lungs, pancreas, and upper gastrointestinal (UGI) tract [4]. We report a case of diffuse gastric ulceration due to radioactive yttrium seed migration to the stomach to alert clinicians to this exceptional cause of gastric injury and to the diagnostic difficulties of this underappreciated and potentially catastrophic complication of hepatic SIRT.

## 2. Case Report

A 57-year-old woman with stage IV breast cancer with liver and lung metastases presented to the hospital with 2 months of worsening intermittent nausea and vomiting. Two months prior, she had received SIRT with ^90^Y resin microspheres. Successful hepatic arterial mapping was done for the planned ^90^Y SIRT. Prophylactic gastroduodenal and right gastric arterial coil embolization were performed without complication. Preliminary scintigraphy was performed through a hepatic artery catheter with Technetium-99m Macroaggregated Albumin (99mTc-MAA). The scan revealed minimal activity in the lungs (7%) with 93% of measured activity in the liver. Yttrium-90 radioembolization of the right and medial segment of the left hepatic lobes was performed without complication. Posttreatment imaging with bremsstrahlung SPECT demonstrated heterogeneous activity in the liver. No significant activity was seen outside the liver including no gastric uptake of microspheres. At the time of SIRT treatment, the patient also had chemotherapy with 5-FU, gemcitabine, cyclophosphamide, and trastuzumab. The patient reported a hospitalization at another institution 4 weeks after SIRT for nausea and vomiting, which were treated symptomatically.

**Figure 1:**
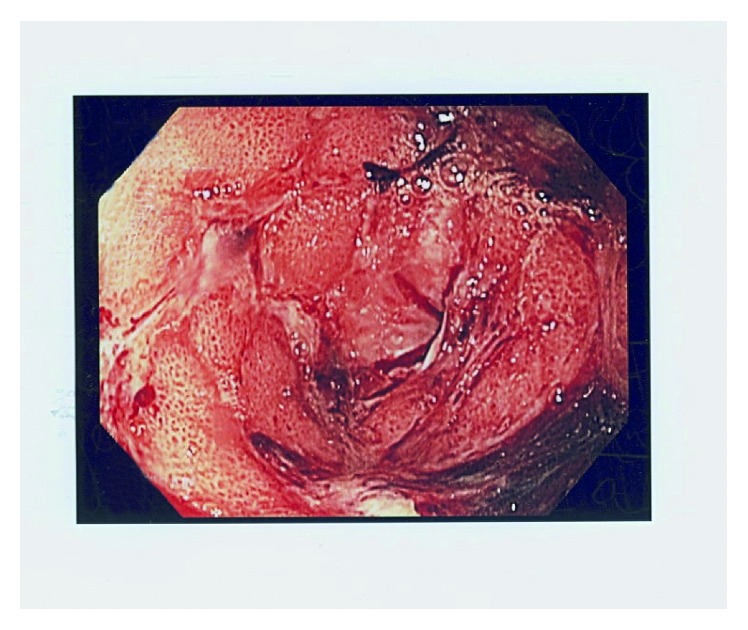
Endoscopic image showing diffuse, severe gastric ulceration.

**Figure 2:**
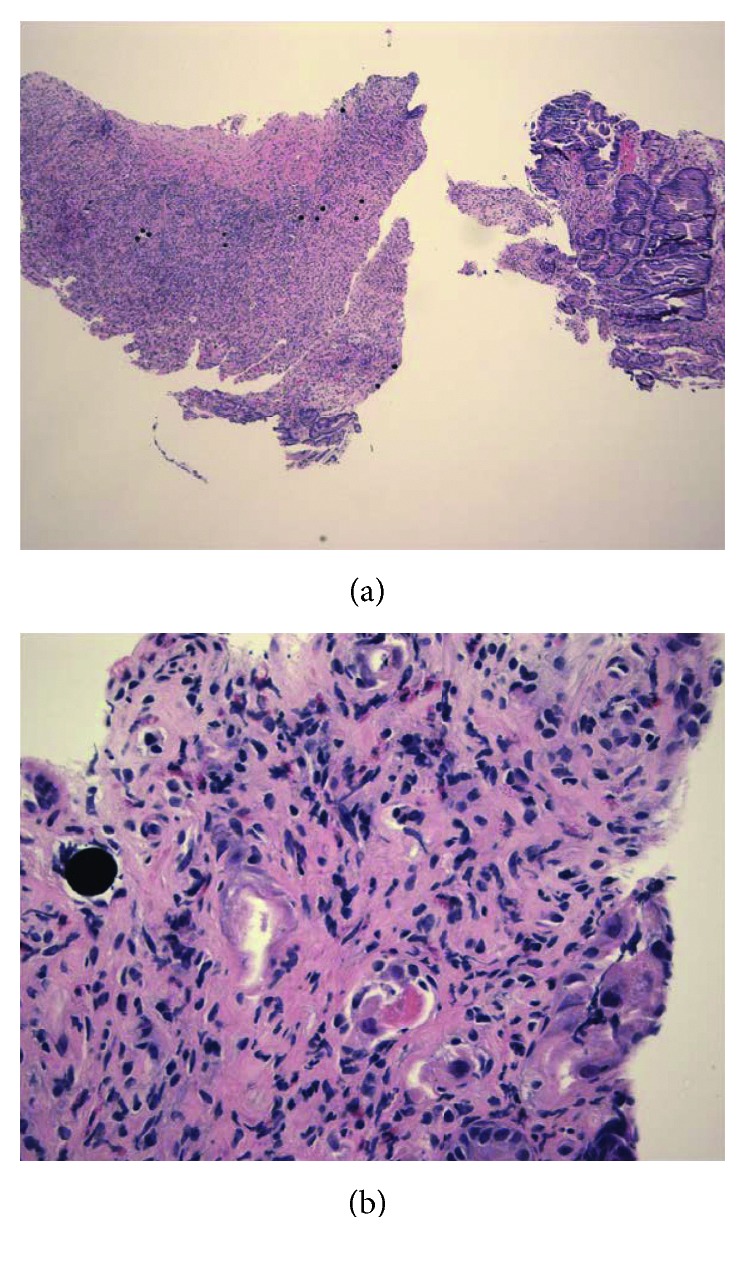
(a) Low-power gastric histology showing multiple black yttrium-90 spheres. (b) High-power histology showing a typical black, gastric yttrium-90 sphere.

On examination at the current hospitalization, vital signs were as follows: blood pressure, 94/51 mmHg (sitting); heart rate, 71/min; O_2_ saturation, 95% on room air; respirations, 20/min; and temperature, 36.6°C (97.9°F). Mucous membranes were dry. Her abdominal exam was without tenderness, organomegaly, or masses. Rectal exam was negative for macroscopic and occult blood. Laboratory data (with normal values in parentheses) revealed a white blood cell count of 5.1 (4.5–11 × 10^9^/L); hemoglobin of 14.4 (12–15.5 g/dl); mean corpuscular volume of 86 (80–96 fL/red cell); platelet count of 158,000 (150,000–450,000/mm^3^); blood urea nitrogen (BUN) of 17; creatinine (Cr) of 0.7 (BUN 6–20 mg/dL; Cr 0.6–1.2 mg/dL); AST of 48 (5–40 U/L); ALT of 35 (7–56 U/L); alkaline phosphatase of 215 (30–120 U/L); total bilirubin of 1.1 (0.2–1.2 mg/dL); albumin of 3.1 (3.5–5.5 g/dL); lipase of 68 (0–160 U/L); and CA 27–29 tumor marker of 599 (normal < 38 U/ml). CT scan of the abdomen and pelvis without contrast showed diffuse gastric antral thickening. UGI endoscopy showed diffuse, severe gastritis and extensive antral ulceration (Figure 1). Maroon blood and superficial duodenal bulb ulcerations were also present; biopsies were taken. On histologic examination, black, spherical foreign bodies consistent with yttrium microspheres were visualized, documenting radiation-induced gastric ulceration. (Figures 2(a) and 2(b)). Biopsies were negative for *Helicobacter pylori*. She received a pantoprazole drip and symptomatic treatment for 72 hours. Since symptoms persisted and her oral intake was insufficient, a jejunostomy tube was placed on the 8th hospital day and enteral nutrition begun. She also received oral steroids. Symptoms improved. She was discharged on the 25th hospital day on a steroid taper, liquid hydromorphone, lorazepam, ondansetron, and pantoprazole.

After discharge, her symptoms did not resolve completely. Repeat UGI endoscopy revealed hemorrhagic gastritis and multiple nonbleeding, gastric antral ulcers with clean bases. Gastric biopsy showed focal gastritis with an inflammatory reaction and degenerative connective tissue consistent with radiation injury. Biopsies of the ulcers were negative for *Helicobacter pylori*. The patient was placed on iron supplementation therapy and pantoprazole. Repeat endoscopy 6 weeks later revealed healing of gastric ulcers.

## 3. Discussion

In selective internal radiation therapy (SIRT), resin microspheres 25–45 *µ*m in size impregnated with radioactive ^90^Y are directly infused into the hepatic arterial circulation [5]. The microspheres become trapped in the tumor microcirculation, releasing targeted beta-radiation [5]. Optimal vascular flow and oxygenation are important in achieving the desired brachytherapy effects and avoiding complications. The average penetration of beta-radiation is 2.5 mm and a maximum of 11 mm of surrounding hepatic parenchyma when used to treat hepatic metastases [4]. The half-life of ^90^Y is 64 hours, with 95% of the dose delivered by day 11 [4]. The tumoricidal effect of radioembolization is predominantly due to radioactivity rather than an ischemia-induced effect [6]. Targeted radioembolization produces shrinkage of tumor size in hepatic metastases from colorectal cancer, in as many as 79% to 91% of patients [4]. Comparable tumor shrinkage has been reported in other metastatic liver malignancies.

**Figure 3:**
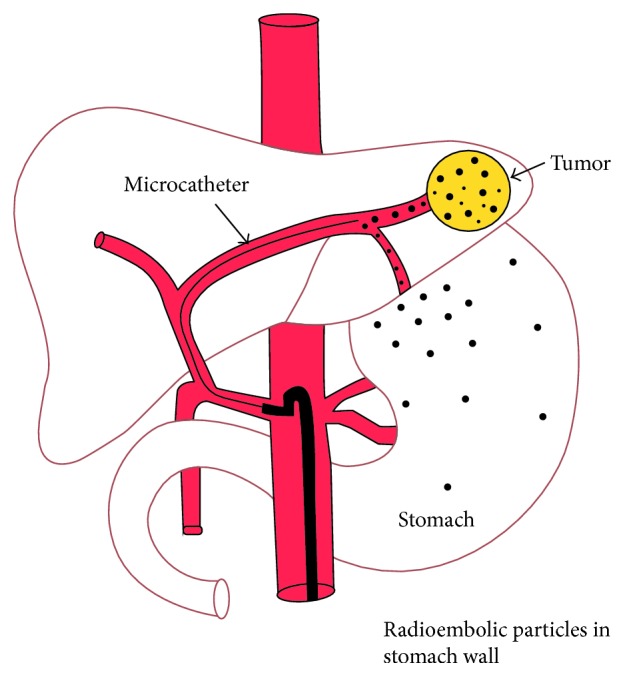
Drawing of aberrant microsphere deposition in the stomach (reproduced with permission of the publisher, *Frontiers in Oncology* [6]).

Yttrium-90 microspheres can cause tissue damage due to aberrant dissemination of radioactivity to nontarget parts of the GI tract or normal hepatic tissue (Figure 3). The reported incidence of GI complications after SIRT for hepatic neoplasia varies widely [7]. Procedure-related morbidity is as low as 5% with strict adherence to contemporary protocols [8]. As in our patient, radiation-induced gastric or duodenal ulceration is caused predominantly by direct mucosal radiation injury from pure beta-radiation. Additionally, mechanical occlusion of arterioles by the 30 *µ*m microspheres can contribute to ischemic injury [1]. The finding that ulcerations typically do not occur after transarterial chemoembolization or when nonradioactive microspheres are injected into the hepatic arteries of experimental animals supports the hypothesis that radiation injury, rather than ischemia, is responsible for these mucosal ulcerations [6].

Gastroduodenal ulceration from nontarget seed distribution to unintended viscera can occur due to variations in hepatic artery anatomy, collateral vessels, or changes in flow dynamics during infusion [8]. A recent root cause analysis indicated that stasis during injection was the strongest independent risk factor for gastroduodenal complications [9]. Knowledge of the hepatic arterial network and its variations is required to identify hepaticoenteric or collateral splanchnic arterial circulation. Pretreatment assessment with digital subtraction angiography and nuclear scintigraphy helps direct specific treatment protocols and minimizes nontarget embolization of radiation [3, 4]. Coil embolization treatment of communicating vessels can be performed if indicated. However, despite meticulous evaluation, these techniques occasionally do not detect small vessels perfusing structures other than the targeted, malignant hepatic tumor. After ^90^Y radioembolization, evaluation of extrahepatic activity and liver dosimetry was performed for our patient by ^90^Y bremsstrahlung SPECT images. However, this technique may have low resolution, which may be another reason for failure to detect sphere distribution in the stomach [10]. As such, some studies have suggested ^90^Y PET as a more accurate method for postprocedure imaging [10, 11].

Typically, as in our patient, the development of ulceration is delayed and has a longer time course than the short half-life of radiotherapy. At times, additional stressors to the gastric mucosa such a toxins, infections, and mechanical trauma may render the stomach more vulnerable to delayed mucosal ulceration. This may impair ulcer healing and may cause scar or adhesion formation [6]. Prior radiotherapy may also inhibit the inherent ability of the mucosa to repair, resulting in a diminished capacity to heal and recover from repeated radiation treatments. Unlike gastric ulcers due to other etiologies that develop at the mucosal surface, ^90^Y-induced ulcers originate from the serosal surface [6].

Delay in diagnosis of gastroduodenal ulcers due to SIRT is common, due to diverse reasons. First, nonspecificity of symptoms such as abdominal pain, nausea, and vomiting mimic more common abdominal or systemic disorders. These symptoms may be vague, insidious, and nonspecific and, thus, attributed erroneously to adjuvant chemotherapy, widespread metastatic disease, or preexisting or new-onset gastroesophageal reflux disease (GERD), thus posing a challenge to timely diagnosis. Second, temporal delay of symptom onset from the time of SIRT administration, as in our patient's two-month delay in symptom onset, lowers the index of suspicion to link these two events. Clinicians must consider radiation-associated toxicity in any SIRT-treated patient developing abdominal symptoms.

Clinicians should also be familiar with the diverse spectrum of multiorgan, acute, and delayed abdominal adverse events after targeted radioembolization to facilitate timely diagnosis and treatment. In addition to the aforementioned symptoms, this treatment can rarely result in cholecystitis, hepatic abscess, decompensation, and liver failure [12]. A postradioembolization syndrome (PRS) can occur, marked by fatigue, nausea, vomiting, abdominal pain of varied severity. Postradioembolization syndrome is common, with reported incidences range from 20 to 70% [1, 6]. In our patient, symptoms of radiation-induced ulceration were delayed, appearing 2 months after the therapeutic procedure.

An important methodological issue in assessing SIRT-related morbidity is that many relevant reports are of single cases or small case series of varying quality and subject to reporting bias. Also, scrupulous adherence to established contemporary protocols associated with a decreased complication rate is not uniformly followed.

Physicians treating patients who have received radioembolization therapy should be familiar with the spectrum of complications and have heightened suspicion for the sometimes delayed, nonspecific adverse effects. Clinicians must consider radiation-associated toxicity in any SIRT-treated patient developing abdominal symptoms.
